# Communism or Christianity?

**Published:** 1894-12-22

**Authors:** 


### COMMUNISM OR CHRISTIANITY?

### A STORY OF THE FUTURE.

The Troubles of the State.

There was no doubt that the winter had set in gloomily. We members of the National Assembly were surrounded with difficulties, knowing that the whole nation looked to us for bread, and that we might have none to give them. I had ventured to make a motion for the reduction of the public rations, but had been defeated. The majority of the assembly agreed with me that it was doubtful if supplies could be kept up to their recent average throughout the winter, now that we could afford to import so little foreign food stuffs, but they dreaded the action of the advanced section, who were always ready to accuse any of the Moderate party of adhering to the old individualist opinions. They in turn dreaded their constituents, who knew nothing of economic conditions and remembered that when the old régime was abolished they had been promised short hours of work, abundance of food, leisure, and amusements—*panem et circenses* to their hearts' content. Short, and even shorter working hours they were likely to have, our trade having diminished to an enormous extent; and amusements could be provided, while the State could force its servants, the actors and singers, to perform in the National Theatre; but where was the bread to come from?

The Individual Destroyed.

Let me briefly describe the situation. Ten years had gone by since the passing of the Act for the Nationalisation of England, by which all estates, mines, factories, warehouses, shops, down to the butcher's stall and the coster's barrow, had been confiscated to the State, and private property had been abolished. Things had at first gone very well on the whole. Some of the old aristocrats and merchant princes had been rusty, but they had for the most part demanded and been granted free passes to the United States of America, where the individualist system still continues; but most of us thought the millennium had come—I might say all, except the Irreconcileable. That was what everyone called him, the old man who lived next door to us in the big model lodging—formerly one of the large hotels—which had been substituted, in order to save labour and promote communism of sentiment, for the old selfish institution of the home. He quoted from some old-world philosopher—Carlyle, I think he was called—a saying that "all the millenniums he ever heard of were prefaced by the chaining up of the devil for a thousand years," and more than hinted that our millennium had begun with the freeing of that personage from such restraints as he had hitherto suffered from. But nobody heeded the Irreconcileable; it was his fashion to grumble. He had some very queer notions, arising to some extent from the fact that he belonged to the sect of Christians, of whom a few still existed, contemptuously tolerated among us. He and I were, after a fashion, friends, though we differed on so many points. He was quite in sympathy with many of our reforms, and therefore I could not quite understand why he condemned others wholesale. "Improve a man's circumstances as much as you choose, but beware how, in doing so, you affect his individuality. You hardly benefit the poorest beggar in the streets, who yet has a soul in him, by making him 'the fattest hog in Epicurus's sty." That was what he said to me the night I came home in triumph to tell him that we had passed the Nationalisation Bill. I did not understand what he meant, and he would give no elucidation. But now I began to have a clearer notion of his views.

The Stimulus of Hope.

As I said, the prospects of the winter were gloomy. The harvest had been bad, and the farmers could not pay their rents. In fact, many of them refused to pay rent at all, and wished to settle down as squatters on the soil. "We thought the landlords were to be abolished," they said, "but it is just the same as ever it was, except that the old landlord took the rent in money, and the State takes our corn and cattle, and leaves us just enough to live on." It was vain to explain that what they paid to the State in one form was returned in another, and that enough to live on was all any man had a right to. They could not understand it, and many thought they were worse off under the new régime than the old. "Then we had the chance of improving our position," said they, "but now it doesn't matter whether we work well or ill; we and ours are none the better off for our exertions." And, apart from the exceptional disaster of this last bad harvest, it was becoming evident that the land was not so well farmed as it was in the old days.

"Can you wonder?" said the Irreconcileable, when I spoke of this to him. "You have deprived them of the stimulus of hope."

"Such hope as that is only selfishness—family selfishness at best."

"But family selfishness is the parent of many virtues—thrift, prudence, energy, self-denial even—all qualities of the first importance to the State."

"Let them be learned directly as virtues, as duties to the state."

"But they can't be. That's the queer thing about human nature. It can't learn a great thing except by means of a small one. When the state ceases to be the development, the natural growth of the family, it is on the verge of disintegration."

"We believe that our state is the family; that is the theory we go upon."

"And you show your respect for it by forcibly substituting your big, unwieldly, man-made, machine-worked Frankenstein family for the smaller, God-created, natural family of father, mother, and child. What have you done?" cried the Irreconcileable. "You meant honestly to improve men's conditions of life, but you forget that to do that permanently you must improve their lives as well; and so you left out half of the machinery—if I must use the diabolical word—necessary to attain your purpose."

Love and Hunger.

Human beings are moved by two impulses, veil them how you will—by love and hunger. You have eliminated love, the divine element, from your scheme of things, and left only hunger, the brutal one."

"Excuse me," I said, "I love my wife."

"True," he answered." "I hope it will not tell against your influence in the National Assembly that you are guilty of such a reactionary weakness. And your wife loves her home—so much of a home as we permit her; loves to exert her fancy in making it dainty, and somewhat cleaner than your state-subsidized housemaids, whom she cannot reprove or dismiss, think worth while. She loves, too, to render you little personal services before you go to work, or when you come home tired—services which I think I have heard you call, before you were married, trivial, and even degrading to womanhood, but which you seem to accept now with pleasure."

That is because they are offered for love's sake. Nothing that love inspires is degrading. The old notion of the wife's duty of service to her husband might be degrading, but I cannot think the same of services rendered for love."

"Thank God for that!" ejaculated the Irreconcilable, "though duty is not such a bad thing either. She manages, too, to fulfil her public work of singing to charm this exacting people of ours, without neglecting you, though sometimes, I fancy, she would be glad to be less tired herself when you come home, to be able to think over the problems that are troubling you and give you her advice and counsel. You do not despise a woman's counsel, I hope?"

"How could I? Have we not made it a fundamental article of our constitution that women have equal powers and rights with men."

"I know; I have not forgotten. But in practice, do you not tend to mistake 'equal' for 'identical'? I value women's influence in the State as much as you; but I think you injure that very influence by teaching that the life and work of home, which should be the training-ground of her nature, are wearisome and degrading. I have read something of the lives of the women who did good work in the old order of things—work which even you acknowledge to have been good—and every one of them had, either by nature or adoption, some of those relations of kinship which develop a woman's nature. Woman is above all things personal; take her out of close relation with some one or two of her kind and she ceases to have any permanent power over the mass of them. Men may be able to settle the hunger problem, but love demands the help of woman, and of the ideal which woman upholds."

"I wish we could settle the hunger problem," I said, "it threatens to become pressing."

"I thought," remarked the Irreconcilable, "that you believed that all the misery of old days was due to over-production, with the inevitable reaction of slack times, and that if you regulated the production we were all to be happy and well-off for ever—especially as there are no highly-paid overseers and other officials, and no capitalists to make a profit out of any business."

A Commonwealth of Laziness.

"I'm afraid," I answered, "that we'll have to institute the overseers again. I thought when we limited the working day to eight hours that the superior energy of the men would result in as much being done in that time as formerly in ten hours. It might be so, I am sure; but it isn't."

"Shall I tell you why?"

"I should be only too glad to know."

"You're not using even your hunger impulse rightly. The workmen don't feel that their daily bread depends on their exertions; it is provided by the State, they think, forgetting that if each man neglects his duty, the State, which is no impersonal entity, but the sum of all these working or idling men, cannot provide food for all. There was a proverb in the old days, "If each would sweep before his own door we should have a clean street." Nowadays nobody sweeps before his own door, and expects the State to clean the street. This is not the case with you and some others—the men who initiated and carried through this change. They were ready to do their own work and that of others as well. Men with heavy burdens of their own, women whose earnestness of soul stood them instead of physical strength, took, like Atlas, the burden of the world upon their shoulders, and tried to move it into a smoother groove than it had ever yet run in. But things run smooth and easily only when going downhill. This world of ours was brought so far along the upward road only by the hard work, the endless pushing and dragging of those who have inhabited it. Aye, oftentimes they have filled up with their own bodies the space that lay between one great step and another. And shall all these fighters and martyrs have spent themselves in vain, like Sisyphus trying to push uphill the stone that always falls again to the bottom? Shall we go down again the dark descent to primeval savagery?"

"Surely not."

"Can you be sure? Are you certain that you will be able to give the next generation as fair a chance as this had? My friend, you may give them leisure, for your trade is not increasing, and your population is; but can you give them bread—that which you have too readily assumed to be the beginning and the end of human needs. You say the workers do not work as they used to do, as you expected them to do. That is not strange; they are not philanthropists and enthusiasts, but men with the faults of their kind. You have emancipated them from the compulsion of individualist greed, you have taken off their shoulders the burden of maintaining themselves and their families, you have made their lives easier, but has it not been at the expense of destroying in them the ennobling influence of individual responsibility?"

"I cannot say," I answered, "I only know that our outlook is gloomy indeed. With our production so small, with our foreign trade gone into the hands of countries which have not adopted the limitation of working hours, with our population increasing faster than ever, I do not know how we can feed the people, at least as we have been doing and hoped to do. We must, at least, limit the expenditure on public entertainments and the decoration of public buildings, We have not money for that."

"And what of the ennobling influence of art on the public mind?"

"We must do without that. After all, bread is the first essential."

### A Premium on Idleness.

We had not foreseen how trade would be affected by the change in our consitution. All manner of complications had arisen. The eight hours limit would not of itself have interfered much, if the work had been maintained at the average of old times, but when bread could be obtained without labour, we found that industry failed. It was so in all classes. The labourer idled because he was assured that he would certainly be able to live more luxuriously than ever before without special exertion on his part; the overseer was indifferent, because no energy on his part could make him better off than the labourer. If the state inspector complained that any factory did not produce as much as it ought, he was condemned as a reactionary, as one of those who demanded that the weakest should keep the level of the strongest, a tyrant, and an individualist in disguise. Pressure was sure to be brought to bear on members of the National Assembly to condemn his action, and he might think himself fortunate if his too great energy did not lead to dismissal. But the production of the country had decreased greatly, and at the same time population was increasing.

### No Personal Responsibility.

This was certainly a direct result of the nationalization. Marriage was no longer limited in the old fashion. A young man had not to strive for years to make a home he thought worthy of his wife. When two persons chose to marry they were at once assigned a lodging, and their share in the public rations went on as before. The State brought up their childen, they had no responsibility in the matter. It had seemed to me at one time a great thing that all mothers should not be made mere nursemaids, that children should be brought up in public *crèches*, but I found that the mothers themselves did not often care to avail themselves of them, and we were forced to leave their employment optional.

"Do you wonder at it?" said the Irreconcileable, when to him I commented on this strange obstinacy. "The child is the mother's dearest self; how can she bear to part with it while it is still helpless, It is bad enough that, as in Sparta of old, it is torn from her for ever when it is seven years old, to be brought up in the State schools."

"Not for ever. Children are restored to their parents at seventeen."

"Yes, when for ten years the child has not known his mother from any other woman; when his parents are as much strangers to him as if their blood did not flow in his veins; when no memory of their care for him in youth dwells with him to make him care for them in old age."

"Parents are not dependent on their children now; the State provides for them," said I.

"It provides them with food," answered the old man; "but can it give them consideration for their weakness, tenderness, honour, love? These things cannot be provided by Act of Parliament."

His words struck me as sentimental folly, excusable from his age, his continual looking backward, and his having long ago, before the days of the nationalisation, lost children whose baby caresses to him, shared as they were with nurses, cats, and dogs, now seemed in memory a much more conscious proof of love than they really were. Yet I could not quite forget what he had said, and when one day I went through a State school I was less satisfied than usual with the appearance of the children. They were healthy, clean, well fed, obedient, not unintelligent; yet something seemed lacking in their expression. I remember having seen in the old days children clinging to their mothers, or running with happy laughter to meet their fathers, and I could not fancy these well-drilled machines giving way to any such emotion.

### The Work of the Good Old Grandmother.

Another point was brought before my notice soon after, which disturbed yet more my conviction that our nationalisation scheme had begun the millennium. One day in a factory I observed a man who was idling, if possible, a little more thoroughly than his fellows. He was a gay, audacious, *insouciant* fellow, whose very appearance irritated me.

"You aren't killing yourself with work, my friend," I said to him with some annoyance.

"No, why should I?" he answered coolly, and with some sarcasm in his tone. "The lives of its citizens are precious to the State, and the State—good old grandmother that it is!—will give me my clothes and my food just as readily if I take things easily as if I take them hard. I didn't do much work under the old system, and I'm not going to wear myself out under the new."

"I expect you were one of the aristocrats," I said severely.

He laughed. "Nature's nobility and that sort of thing, if you like; but the vulgar called me a tramp."

"Did you never do a hard day's work?" I asked.

"Oh, yes, a long time ago, before my wife died. After that there was no one to work for."

"There is the nation. Do you value your whole country less than one woman?"

"Wait till your wife dies and you'll see," he answered curtly, and took up his work with more energy than he had yet displayed.

His words clung to me insistently, and for no other reason than that my wife had been ailing that morning when I left home. I had to go through my hours of work, but then I hurried home at my best speed. "My wife, how is she?" I demanded of the doctor, who was the first person I met.

"There is no cause for anxiety," he answered. "Your child is born and is healthy, and your wife is wonderfully well. You can see her for a few minutes."

"Thank God!" I exclaimed, for the words of the old creed seem still to rise instinctively to my lips in moments of emotion.

When I entered her room, my wife, lying on her couch, pale, languid, and beautiful, smiled to me. As I bent over she uncovered with her white transparent hand the face of the sleeping child by her side.

"Is he not beautiful ?" she whispered, mother-like. Then a fierce look came into her eyes, so soft and gentle a moment before. "But he is mine—mine and yours—and they are not to take him from us. We will go to some place where they cannot claim him."

I took her words for the mere outcome of a weakness which at the moment approached delirium. But when she regained strength the idea still remained continually present with her—the longing to get away to some place where the State could not claim her child.

Husband," she said, "can we not emigrate ? Can we not go to some country where the State will not come between us and our child ? You are strong enough and clever enough to earn bread for us; I will save and pinch—ah ! what can I not do if my child is not taken from me !"

Give us Our Wings Again.

"You know how emigration is discouraged," I told her. "It was a cruel necessity in the old days, when food could not be found for all, and families left their homes and crossed the seas to find it; but now we hope there will be no need for that."

"People emigrated for freedom's sake as well. We have food now, but we have not freedom. I would fain go to some country where the State does not interfere between parent and child."

"You know how often before our present law was passed the State was forced to protect the child from the parent."

"Yes, but in all the bad parents were few compared with the good. And it is one thing for the State to interfere where the law of nature seems to fail, another to substitute an artificial routine for a natural order. The State should be the administrator of natural laws, taking care that the rights of one man do not interfere with those of another. It should not substitute external machinery for the impulses of nature."

"Yet," I said, "many of our women seem to be indifferent to parting with their children. It leaves them more free for work and pleasure."

"Ah, poor things !" said my wife, "how can they learn to be mothers without children to teach them ? How are they to learn duty when everything is done by deputy ? I think, husband, that in trying so much to lighten every man's burden, our State has forgotten that things grow only by exercise. One does not strengthen oneself by placid walking along a street so much as by climbing a hill. I think we have tried too much to banish effort, pain, and exhaustion from our lives, and the end is that, at least among our women, those are the noblest and the happiest who have volunteered to spend their lives in tending the sick."

"You have been talking to the Irreconcileable," I said.

"Sometimes he talks to me," she admitted. "He tells me of old times which I can scarcely remember, and I love to think of them."

"There was plenty of pain and sorrow in the old days; poverty and anxiety such as we cannot realize now."

"I know," she said; "but there was hope, there was energy, there was aspiration. One could believe that men had souls in those days, because they suffered and rebelled; because, defeated or victorious, they had something to strive for, something to attain. But we—we are fed like the beasts of the field, and, like them, have neither past nor future. Formerly some might be in that condition, but now we are all alike."

I warned her that she had better not express these opinions too openly, or she might find that in our present system, if there was no hope, there was, at least, suffering for those who rebelled.

I, too, talked to the Irreconcileable—the only person with whom I dared discuss, even as a possibility such a project as this of leaving the country in order to evade giving up our child to the national guardians.

"I dare not—I who helped to bring about this freedom—own that I regret in any point the individualistic selfishness and tyranny of the past."

"Tyranny !" grumbled the old man. "Is there any worse tyranny than that which denies to a man and a woman any property in their own child ?"

Memories of an Old Religion.

"Yes," I said, for we were now in the dark days of December, "I remember about this time an old-fashioned festival in honour of the birth of a child who gave Himself up for the redemption of the world."

"Yes," he answered, "but you forget that for thirty years that child was the sunshine, perhaps the mainstay, of a village home before ever the world heard of Him, or accepted His sacrifice. You, and those who think like you, ignore the divine mystery of growth. 'First the blade, then the ear, then the full corn in the ear,' as He Himself states it. You grow from babe to man, from the lesser to the greater. First the home, then the wider circle of friends and kindred, then the neighbourhood, all teaching their lessons and bringing their discipline before the man is fit to take his part in the community at large. And in true growth each stage will lead insensibly to the other, and be linked to it for ever. Trust me, if Mary of Nazareth had not seen or known her Son since His childhood she would not have had the courage to kneel at the foot of His cross, nor would He in His agony have had a thought to give to the care and protection of her old age. The divine soul, which alone makes man better than the brutes, can only be developed by the divine institutions of love, parenthood, and kinship."

I answered nothing, for indeed I could not but see that around me there was as much coldness and selfishness as ever, though it had changed its character. The old family selfishness was killed, but I doubt if the lazy personal selfishness which replaced it was of a higher type.

Eventually my wife's entreaties, aided by the old man's arguments, persuaded me, and I resolved to leave the country with my family. The Irreconcileable's face beamed when I told him of our decision.

"You ought to come with us," I said to him, "for it is largely by your advice that we have been guided."

The Machine-made Nation.

He shook his head. "If it were leaving only this machine-made nation I should not hesitate, but I should have to leave the graves of those I have loved, the most sacred treasure I now possess. And even amidst the machinery I may find other human souls, like yours, protesting against this artificial nation-making. As for you, you have now a better teacher than I. This Christmas-tide has taught you the real Christmas lesson—the value of childhood, the sacredness of family love." And he quoted from his old-world Scriptures—"A little child shall lead them."

### "TRUTH'S" TOY EXHIBITION.

The Annual Exhibition of Toys in the Royal Albert Hall, under the auspices of *Truth*, possessed a special interest this year from the "competitions" connected with the 3,000 dolls dressed by readers of the paper. The show itself, as brilliant a success as usual, will result in the distribution of toys to some 27,000 children in workhouses, hospitals, and infirmaries.

